# Tissue penetration of anti-tumour necrosis factor therapy in perianal fistulising Crohn’s disease: a proof-of-concept study

**DOI:** 10.1097/MEG.0000000000003169

**Published:** 2026-02-24

**Authors:** Sulak Anandabaskaran, Zhigang Liu, Luke Hanna, Phillip Lung, James L. Alexander, Nick Powell, Susan J. Connor, Phil Tozer, Ailsa Hart

**Affiliations:** a School of Clinical Medicine, University of New South Wales Faculty of Medicine, Sydney, New South Wales, Australia; b Department of Metabolism, Digestion and Reproduction, Imperial College London; c Robin Phillips Fistula Research Unit, St Mark’s the National Bowel Hospital, London; d Inflammatory Bowel Diseases Unit, St Mark’s Hospital, Harrow; e Department of Gastroenterology, Imperial College Healthcare NHS Trust, London, UK; f Department of Gastroenterology, Liverpool Hospital, Liverpool, New South Wales; g South West Sydney Clinical Campuses, UNSW Medicine & Health, UNSW Sydney, Sydney, Australia

**Keywords:** adalimumab, drug levels, infliximab, outcomes, perianal Crohn’s disease

## Abstract

**Background:**

Perianal fistulising Crohn’s disease (pfCD) remains a therapeutic challenge, with a limited sustained response to biological therapy. Although higher serum anti-tumour necrosis factor (TNF) levels are associated with improved fistula healing, tissue pharmacokinetics in pfCD are poorly understood. This proof-of-concept study aimed to establish the feasibility of quantifying anti-TNF concentrations within fistula tissue and evaluate their relationship with serum levels and treatment outcomes.

**Methods:**

Paired blood and fistula tract biopsies were obtained from 14 patients (infliximab, seven; adalimumab, seven) with active pfCD on established anti-TNF therapy (>14 weeks post-induction). The serum was processed by centrifugation within 8 h and stored at −80°C. Fistula tract biopsies were snap-frozen, homogenised, and extracted using an ELISA buffer proportional to tissue weight. Anti-TNF levels in the serum and tissue supernatants were quantified using standard and high-sensitivity ELISA assays, respectively.

**Results:**

All patients had detectable anti-TNF concentrations in both serum and fistula tissues. Tissue and serum levels showed a moderate positive correlation (*r* = 0.45, *P* = 0.09), with a stronger and statistically significant association in the infliximab subgroup (*r* = 0.81, *P* = 0.01). Higher fistula-to-serum ratios, reflecting enhanced tissue penetration, tended towards improved clinical and radiological outcomes and lower perianal disease activity index scores, although the difference was not statistically significant.

**Conclusion:**

Anti-TNF levels in perianal fistula tissue are measurable and correlated with serum concentrations, supporting a mechanistic link between systemic exposure and local drug penetration. These findings highlight the feasibility of tissue-level pharmacokinetic assessments and warrant validation in larger prospective cohorts.

## Background and aims

Perianal fistulising Crohn’s disease (pfCD) is a severe, chronic, relapsing-remitting phenotype of inflammatory bowel disease (IBD) that affects up to 30% of individuals with Crohn’s disease [1,2]. It is associated with significant morbidity, debilitating symptoms, and a markedly impaired quality of life [2,3]. The current immunopathogenic model suggests a dysregulated inflammatory response driven by pro-inflammatory cytokines, most notably tumour necrosis factor-alpha (TNF-α), which is expressed in perianal fistulas [4]. This model is supported by high-quality evidence demonstrating the efficacy of anti-TNF agents as first-line medical therapy for complex pfCD, endorsed by both the ECCO and BSG guidelines and the TOpClass consortium [5–7].

Infliximab and adalimumab are the most widely used anti-TNF agents in this context. These monoclonal IgG1 antibodies bind to TNF-α with high affinity, neutralising its activity and preventing downstream inflammatory signalling [8]. Despite this targeted approach, long-term response rates remain modest, with clinical remission achieved in only 40–50% of patients and healing on MRI in less than 10% of patients [9–11]. Retrospective studies also suggest that higher serum trough levels of infliximab and adalimumab are associated with improved outcomes [12,13]. Nevertheless, nearly half of the initial responders lose response within the first year of treatment [3,14], likely due to multifactorial causes, with anti-drug antibody formation playing a key role [3]. In addition, the efficacy of anti-TNF agents in luminal disease control is greater than that in perianal disease control, with some patients responding well from a luminal point of view but less so or not at all in terms of their perianal disease, despite adequate serum drug levels. This raises the question of tissue-level drug availability and the possibility that systemically administered medications simply do not penetrate perineal tissues.

Local intra-fistula injections of anti-TNF agents have been trialled in small pilot studies, but the results have been mixed, and long-term efficacy remains unclear [15]. Given the deep extracellular matrix remodelling characteristics of pfCD, a better understanding of tissue drug penetrance is essential [4].

Evidence from luminal Crohn’s disease further illustrates this. Yarur *et al.* [8] used a homogeneous mobility shift assay to demonstrate that tissue levels of anti-TNF drugs correlate well with serum concentrations. Notably, severely inflamed areas acted as a ‘sink’, absorbing more of the drug – a phenomenon that may explain why some patients experience active disease despite therapeutic serum levels [3,8]. The authors suggested that patients with extensive inflammation might require higher drug doses to achieve adequate concentrations at the disease site.

Building on this concept, Adegbola *et al.* [3] assessed tissue anti-TNF levels in patients with pfCD. Liquid chromatography-mass spectrometry (LC-MS) was used to examine biopsies from perianal fistula tracts. Although the assay detected infliximab and adalimumab in spiked controls, no drug was identified in actual tissue samples [3]. The authors proposed that this may reflect either inadequate drug penetration into the fistula tissue or increased local consumption of the drug due to the high inflammatory burden, but may also reflect limitations of LC-MS sensitivity. Given these potential technical constraints, alternative assays are needed. ELISA is well-validated and widely used in routine clinical practice to measure serum anti-TNF levels [16].

In light of these findings, we aimed to determine whether tissue anti-TNF levels in pfCD can be accurately measured using an ELISA and whether they correlate with serum concentrations. Finally, we evaluated whether tissue penetration of anti-TNF drugs predicts treatment response and long-term outcomes in pfCD, thereby addressing a critical gap in therapeutic drug monitoring by extending it from serum to tissue levels.

### What is already known?

- Therapeutic drug monitoring in Crohn’s disease focuses on serum anti-TNF levels.
- Patients with pfCD are often refractory to anti-TNF therapy, despite therapeutic serum levels.
- Prior attempts to measure tissue anti-TNF levels in pfCD to further understand direct anti-TNF tissue pharmacokinetics using an LC-MS assay were unsuccessful.

### What is new here?

- This is the first study to quantify anti-TNF drug levels in perianal fistula tissues using ELISA.
- Fistula tissue anti-TNF levels correlate well with serum anti-TNF levels.
- Higher tissue-to-serum ratios trend towards better clinical and radiological outcomes.

### How can this study help patient care?

- Demonstrating measurable anti-TNF levels in fistula tissue supports the extension of therapeutic drug monitoring from the serum to the tissue.
- Understanding tissue drug penetration may offer valuable insights and enhance our understanding of IBD pharmacotherapy.
- In turn, improving treatment optimisation, individualised dosing, and outcomes in refractory pfCD.

## Materials and methods

**Fig. 1.**
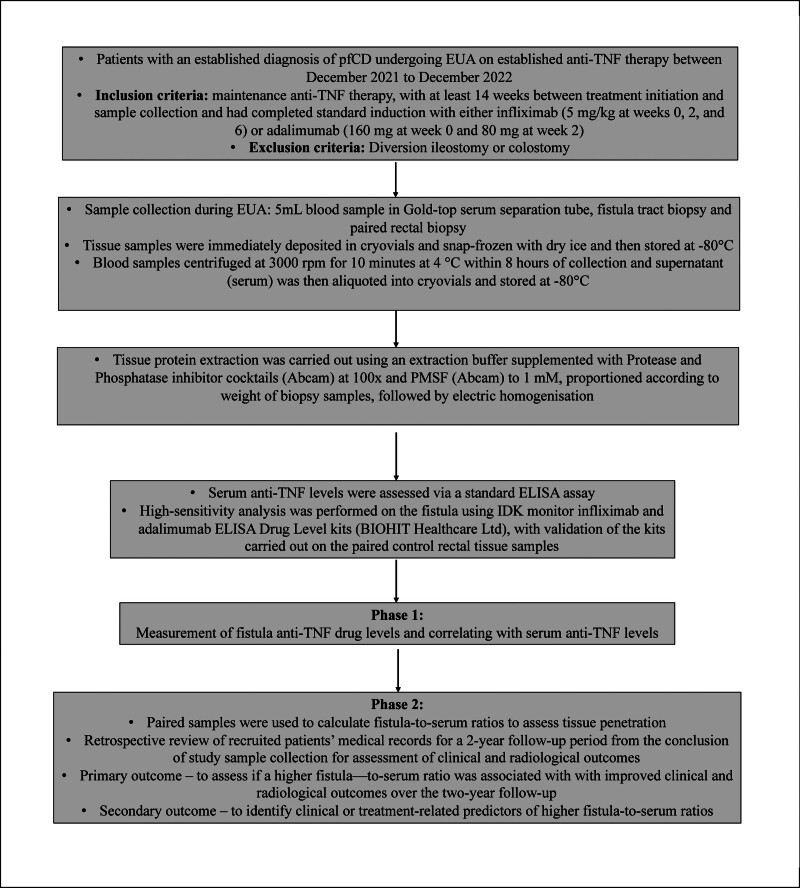
Methodology diagram. EUA, examination under anaesthesia; pfCD, perianal fistulising Crohn’s disease; TNF, tumour necrosis factor

This was a single-centre prospective cross-sectional study of patients undergoing examination under anaesthesia (EUA) and on established anti-TNF therapy for Crohn’s perianal fistula(s). This study was approved by the NHS Health Research Authority (22/WA/0214/R20025; sub-collection ID: SMT_PT_20_001). Patients were enrolled over 12 months, from December 2021 to December 2022, when they presented for EUA and met the inclusion criteria (Fig. 1). This was an observational study embedded within routine clinical care; hence, EUAs were not protocolised to coincide with specific anti-TNF dosing time points. Patients were included if they were receiving maintenance anti-TNF therapy, with at least 14 weeks between treatment initiation and sample collection, and had completed standard induction with either infliximab (5 mg/kg at weeks 0, 2, and 6) or adalimumab (160 mg at week 0 and 80 mg at week 2). Maintenance dosing was intravenous for infliximab and subcutaneous for adalimumab. All maintenance dosing schedules of infliximab and adalimumab were considered eligible to capture the real-world variability in treatment regimens. The dosing decisions were made at the discretion of the treating gastroenterologist. Patients who underwent diversion ileostomy or colostomy were excluded from the study.

Written information was provided, and informed consent was obtained in accordance with research ethics guidelines. Patient demographics, including age, sex, medical history, patient-reported perianal disease activity index (PDAI; scored out of 12), medication use (including concomitant immunosuppression), and historic trough level (within 2 years of sample collection), were collected through patient questionnaires and reviews of online medical records on the day of EUA.

Paired blood and tissue samples were collected during EUA. Blood (5 ml) was collected in a Gold-top Serum Separation Tube (BD Vacutainer, Fisher Scientific, Leicestershire, UK) during cannulation. A fistula tract biopsy was performed from the central aspect of the fistula using a surgical scalpel, and the fistula tract was non-epithelialized. Four paired rectal biopsies were also collected using endoscopy forceps to act as non-fistulising control tissue for validation of the ELISA assays but were not included in the primary analyses. Fistula tract and rectal biopsies were then immediately deposited in cryovials, snap-frozen with dry ice, and stored at −80°C until supernatant extraction. Blood samples were incubated at room temperature for no longer than 8 h and then centrifuged at 3000 rpm for 10 min at 4°C. The supernatant (serum) was then aliquoted into cryovials and stored at −80°C until drug level measurements were performed.

For tissue protein extraction of anti-TNF, biopsy samples were thawed on ice before supernatant extraction. The tissue samples were weighed and transferred to sterile tubes. For every 5-mg piece of tissue, 300-μl complete ELISA extraction buffer (100 mM Tris pH 7.4, 150 mM NaCl, 1 mM EGTA, 1 mM EDTA, 1% Triton X-100, 0.5% sodium deoxycholate) supplemented with protease and phosphatase inhibitor cocktails (Abcam, Cambridge, UK) at 100× and PMSF (Abcam) to 1 mM were added and homogenisation was carried out with an electric homogeniser. Samples were then maintained under constant agitation for 2 h at 4°C by placing them on an orbital shaker. Following this, they were centrifuged for 20 min at 13 000 rpm at 4°C. Samples were then placed on ice, and supernatants were aliquoted into cryovials and stored at −80°C until drug level measurement was carried out. Preliminary analysis through the Exeter Clinical Laboratory identified serum anti-TNF levels using a standard ELISA assay [17,18]. However, this failed to identify anti-TNF levels across all tissue samples, despite successfully detecting serum levels. Therefore, a secondary high-sensitivity analysis was performed on the fistula and control rectal tissue samples according to the manufacturer’s instructions using IDK monitor infliximab and adalimumab ELISA Drug Level kits (BIOHIT Healthcare Ltd., Bensheim, Germany) [19,20].

This study was conducted in two phases (Fig. 1). In Phase 1, the primary outcome was to establish whether fistula anti-TNF drug levels could be reliably measured using an ELISA assay. Tissue anti-TNF levels (ng/ml) were normalised to biopsy mass by dividing the measured concentration by the tissue weight (mg), yielding weight-adjusted values expressed as ng/ml per mg of fistula tissue. The secondary outcome of phase 1 was the correlation between fistula tissue and serum drug levels.

In Phase 2, paired fistula and serum samples collected at the same time point were used to calculate fistula-to-serum ratios as a marker of tissue drug penetration. Recruited patients’ medical records were also retrospectively reviewed for a 2-year follow-up period (until December 2024), from the conclusion of the study sample collection, to obtain clinical and radiological improvement outcomes. The primary outcome was whether higher fistula-to-serum ratios (improved tissue penetration) were associated with improved clinical and radiological outcomes over the 2-year follow-up. Clinical outcomes were assessed by TOpClass improvement and radiological response on pelvic MRI performed 12–24 months after sampling, according to TOpClass radiological criteria [21]. The 12- to 24-month MRI window was chosen to capture medium-term radiological response attributable to tissue drug exposure while limiting confounding by subsequent therapeutic interventions. The secondary outcome was the identification of the clinical or treatment-related predictors of higher fistula-to-serum ratios. Statistical analyses were performed using GraphPad Prism version 10.5.0. Most variables were measured on a continuous scale, and associations were examined using correlation methods. Pearson’s correlation was applied when variables were approximately normally distributed, whereas Spearman’s correlation was used for variables with outliers. Comparisons between the patient subgroups were performed using unpaired *t*-tests for normally distributed continuous variables.

Mann–Whitney tests were used for continuous variables with outliers, and Fisher’s exact tests were used for categorical variables. Fisher’s exact test was used to compare categorical outcomes between the groups.

## Results

Fourteen patients receiving anti-TNF therapy, with matched serum and fistula drug levels, were included in the final analysis. Patient demographics are summarised in Table 1.

**Table 1.** Patient demographics

Demographics	Total (*n* = 14)
Age, mean, y ± SD	36.3 ± 15.1
Male sex, *n* (%)	7 (50.0%)
Race	
Caucasian, *n* (%)	12 (85.7%)
Asian, *n* (%)	2 (14.3%)
Weight, mean, kg ± SD	72.1 ± 11.7
Smoking	0 (0.0%)
Disease characteristic	
Duration of CD, median, months [IQR]	60.5 [34.3, 92.3]
Duration of PAF, median, months [IQR]	34.5 [21.0, 62.3]
Prior perianal fistula surgery, *n* (%)	12 (85.7%)
Seton in-situ, *n* (%)	10 (71.4%)
PDAI, mean ± SD	5.5 ± 1.7
Anti-TNF therapy	
Infliximab, *n* (%)	7 (50.0%)
Adalimumab, *n* (%)	7 (50.0%)
Duration of current anti-TNF therapy, median, months [IQR]	20.5 [9, 51]
Concurrent medical therapy	
Immunomodulator, *n* (%)	8 (57.1%)
Corticosteroids, *n* (%)	0 (0.0%)
Antibiotics, *n* (%)	1 (6.7%)
Montreal classification	
Age at time of diagnosis	
A1 (≤16 years), *n* (%)	4 (28.6%)
A2 (17–40 years), *n* (%)	7 (50.0%)
A3 (>40 years), *n* (%)	3 (21.4%)
Location	
L1 (terminal ileum), *n* (%)	4 (28.6%)
L2 (colon only), *n* (%)	7 (50.0%)
L3 (ileocolonic), *n* (%)	3 (21.4%)
L4 (upper GI), *n* (%)	0 (0.0%)
Behaviour	
B1 (non-stricturing, non-penetrating), *n* (%)	12 (85.7%)
B2 (stricturing, non-penetrating), *n* (%)	1 (7.1%)
B3 (penetrating), *n* (%)	1 (7.1%)
Parks classification of fistula anatomy^a^	
Intersphincteric, *n* (%)	6 (42.3%)
Transphincteric, *n* (%)	11 (78.6%)
Suprasphincteric, *n* (%)	1 (7.1%)
Extrasphincteric, *n* (%)	2 (14.3%)
TOpClass classifications	
Class 2a, *n* (%)	2 (14.3%)
Class 2b, *n* (%)	11 (78.6%)
Class 2c-i, *n* (%)	1 (7.1%)

IQR, interquartile range; PDAI, perianal disease activity index.

a Patients may have >1 fistula tract classification.

### Phase 1

**Fig. 2.**
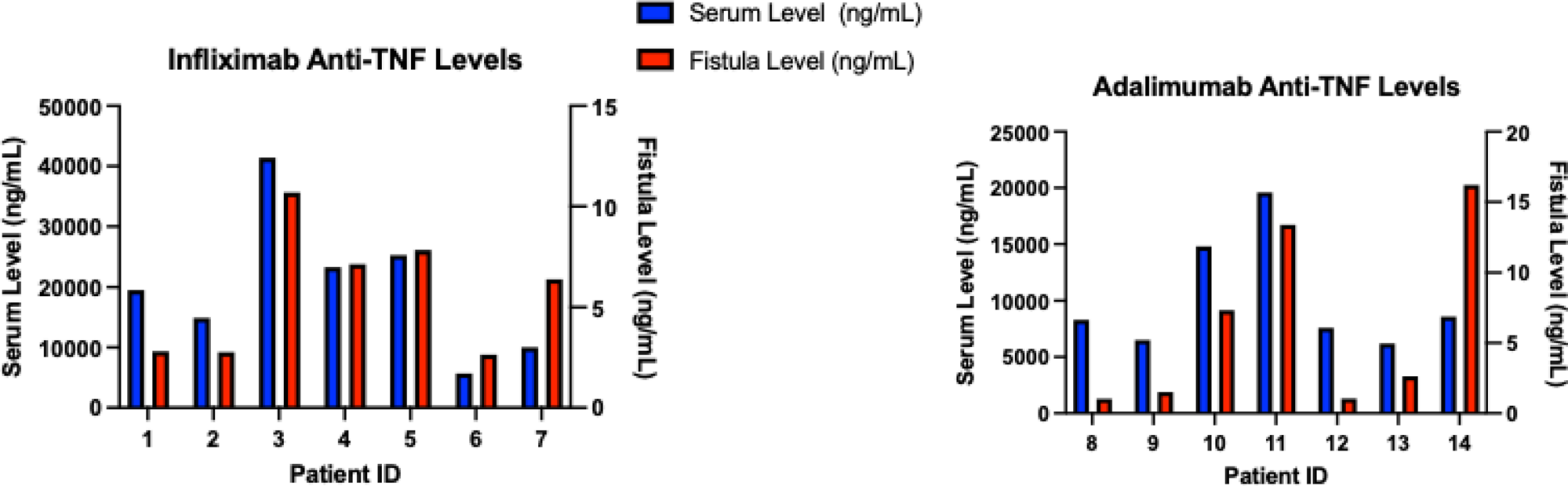
Respective serum anti-TNF levels and fistula anti-TNF levels (per mg of tissue) in the infliximab and adalimumab groups. TNF, tumour necrosis factor.

All patients had detectable serum anti-TNF levels as determined by standard ELISA. Fistula anti-TNF levels were also successfully detected in all 14 patients, and drug levels were similarly detectable in paired rectal control tissues collected for validation of the high-sensitivity ELISA. Figure 2 shows the respective serum and fistula anti-TNF levels of all patients. Anti-TNF levels in fistula tissue correlated positively with serum levels (*r* = 0.44, *P* = 0.12). Sub-analysis by drug type revealed similar trends, with a significant positive correlation between serum and fistula anti-TNF levels in the infliximab subgroup (*r* = 0.80, *P* = 0.03), whereas no statistically significant association was seen in the adalimumab group (*r* = 0.58, *P* = 0.17).

### Phase 2

**Fig. 3.**
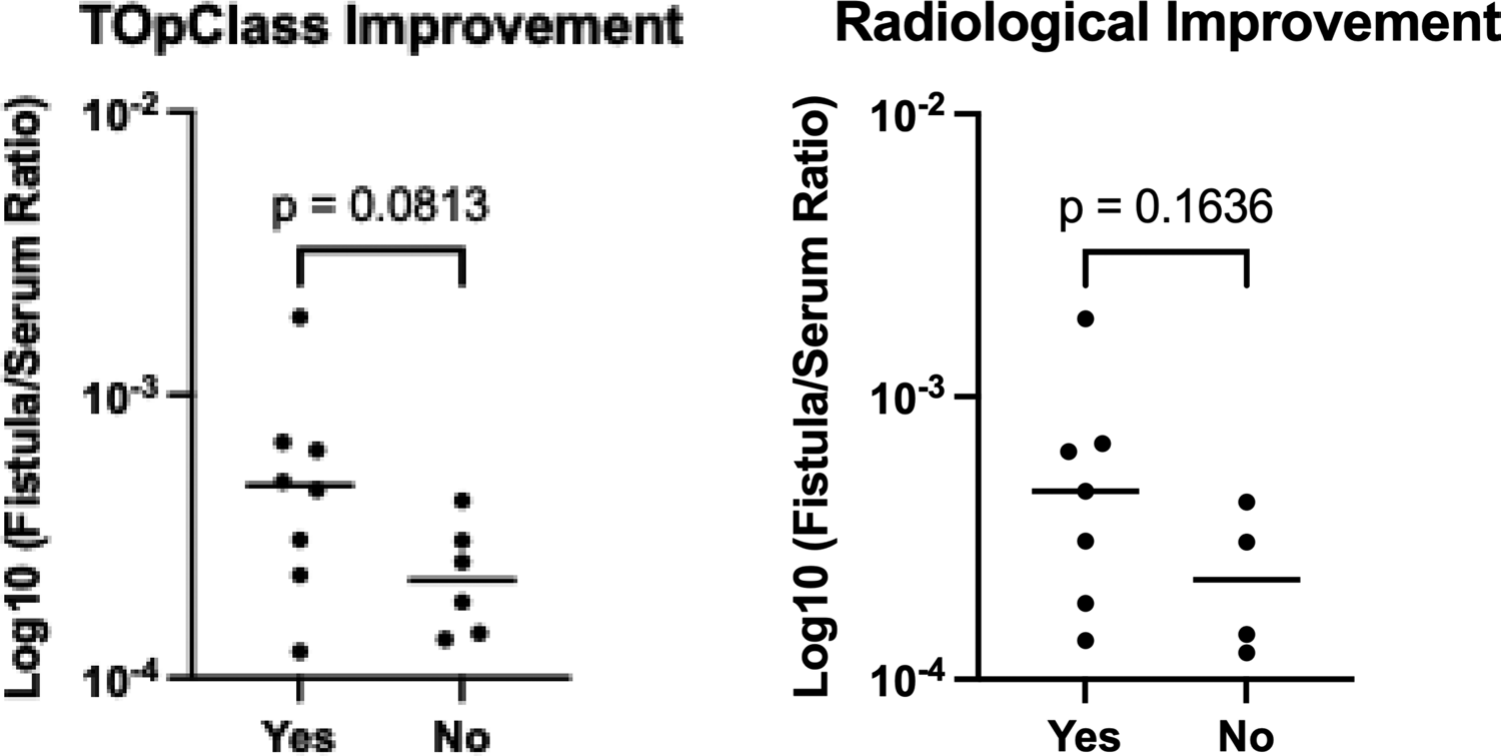
Patients who demonstrated clinical improvement, defined by an improvement in TOpClass classification at study conclusion, tended to have a higher median fistula-to-serum ratio. Among patients with available follow-up pelvic MRI (*n* = 11), those with radiological improvement defined by TOpClass criteria also exhibited higher median fistula-to-serum ratios.

Given that all patients had paired detectable fistula and serum anti-TNF levels, we were able to calculate fistula-to-serum ratios as markers of tissue drug penetration. Better tissue penetration was associated with a trend towards more favourable treatment outcomes; however, these did not reach statistical significance, likely due to the small sample size. The PDAI score was negatively correlated with the fistula-to-serum ratio (*r* = −0.25, *P* = 0.39), suggesting that reduced tissue penetration may be associated with a greater symptom burden. Follow-up pelvic MRI within the predefined 12- to 24-month window was available for 11 of 14 patients. The median time from sample collection to MRI was 16 months (IQR 14–20). Higher fistula-to-serum ratios showed a trend towards improved downstaging of the disease according to the TOpClass classification (*P* = 0.08) and radiological improvement (*P* = 0.16) at 2 years (Fig. 3).

**Fig. 4.**
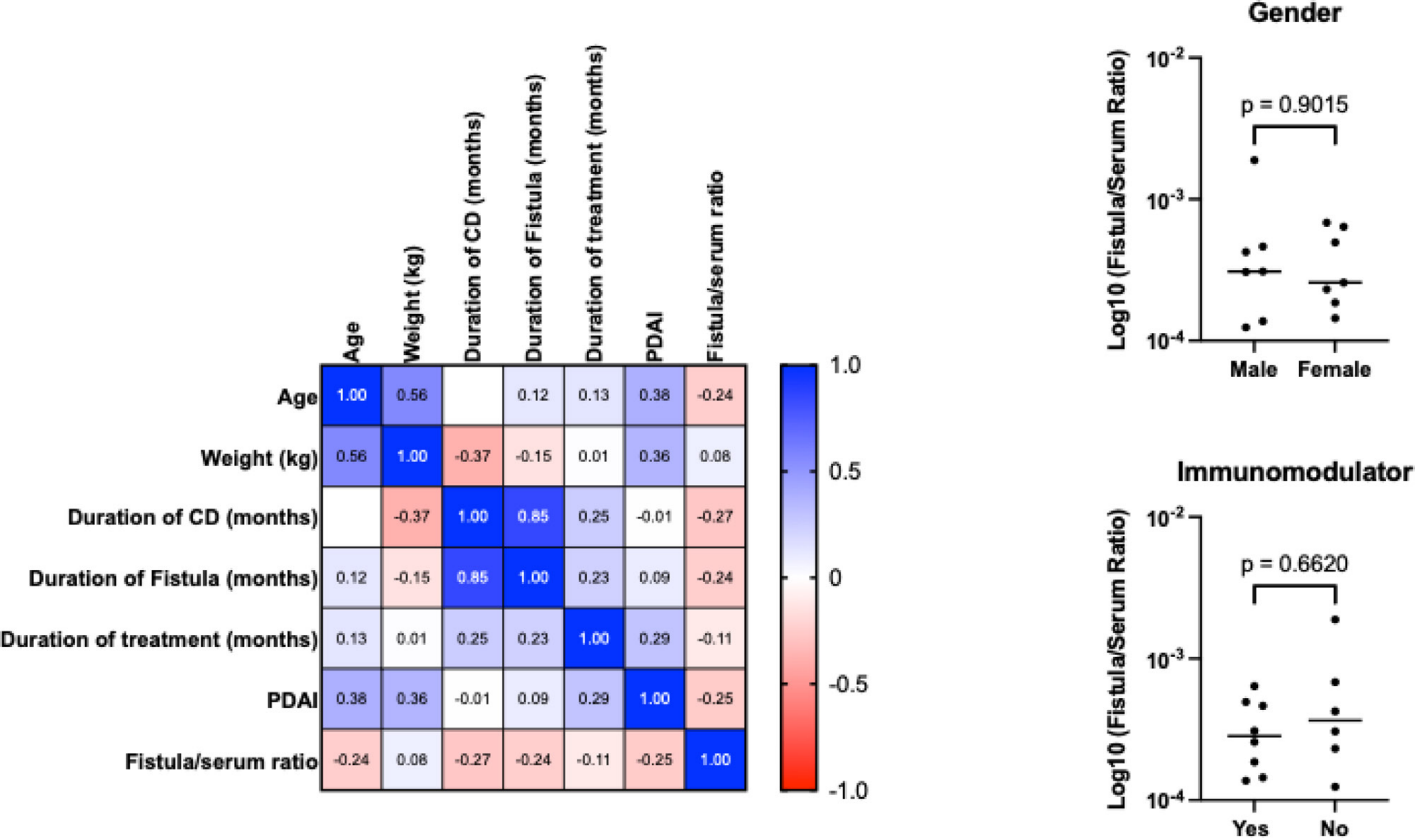
No significant correlations were observed between fistula-to-serum ratios and patient demographic or treatment-related factors. PDAI, perianal disease activity index.

No significant correlation was observed between patient demographics or treatment-related factors and fistula-to-serum ratios (Fig. 4). Categorical baseline factors, including sex or immunomodulator use, were also not associated with improved tissue penetration with similar fistula-to-serum ratios (Fig. 4). However, the most recent historical trough level available (*n* = 9) before tissue sampling showed a non-significant but positive correlation with the fistula-to-serum ratio (*r* = 0.54, *P* = 0.14).

## Discussion

In this proof-of-concept study, we demonstrated for the first time that anti-TNF drug levels in perianal fistula tissue are both measurable and correlate well with serum levels. This underscores the potential importance of direct tissue pharmacokinetics for understanding the treatment response in pfCD [22]. While overall serum and fistula concentrations showed a moderate positive association, a statistically significant correlation was observed only within the infliximab subgroup. Although this finding may suggest differences in tissue drug penetration between infliximab and adalimumab, interpretation is limited by small subgroup sizes, variable dosing intervals, and non-standardised sampling, and may therefore reflect timing or assay-related variability rather than true pharmacokinetic divergence. Nevertheless, recent evidence from a systematic review and meta-analysis indicates that adalimumab is not inferior to infliximab in the treatment of pfCD [23], reinforcing the need for larger studies, such as ours, to further delineate potential tissue pharmacokinetic and clinical differences between these agents.

Our findings extend prior work that has informed luminal IBD [8,24,25] and pfCD [10,12,26] guidelines, as well as the recently published TOpClass guidance [7], all of which emphasise the importance of achieving higher serum levels in pfCD. Here, we provide mechanistic evidence that higher systemic anti-TNF exposure translates to greater local drug penetration within fistula tissue, potentially leading to improved outcomes.

Importantly, lower fistula drug levels tended towards higher PDAI scores, consistent with the hypothesis that insufficient local tissue exposure contributes to persistent symptom burden. The observed trend towards improved clinical and radiological outcomes among patients with higher fistula-to-serum ratios further supports a mechanistic link between tissue drug penetration and disease control. Although not statistically significant, the moderate positive association between historic trough levels and fistula-to-serum ratio suggests that sustained higher systemic exposure may enhance drug penetration into the fistula tissue; however, these trough measurements were temporally separated from tissue sampling and may not fully reflect drug exposure at the time of examination under anaesthesia, particularly in the context of interval dose adjustments. This observation nevertheless aligns with the broader therapeutic drug monitoring literature, in which higher serum anti-TNF concentrations have been associated with improved fistula healing [10,12]. From a clinical perspective, these findings raise the possibility that tissue-level drug assessment could help inform surgical decision-making, including the timing of seton removal or definitive fistula closure. Prospective studies incorporating protocolised tissue sampling relative to dosing intervals and predefined surgical milestones will be required to determine whether optimisation of tissue drug penetration prior to surgical intervention translates into improved radiological healing and durable fistula closure.

The limitations of this study should be acknowledged, particularly those related to tissue methodology and sample size. Perianal fistula tissue is inherently heterogeneous and comprises variable proportions of granulation tissue, fibrosis and inflammatory infiltrates. To minimise sampling variability, biopsies were consistently obtained from the active granulation zone using a standardised technique previously applied in our transcriptomic analyses, in which inflammatory markers were reliably detectable [27,28]. Tissue extraction was standardised by weighing each biopsy and normalising the extraction buffer volume per milligram of tissue to ensure proportional drug recovery. The standard Exeter ELISA assay was unable to detect anti-TNF in tissue because of its high lower limit of detection (~800 ng/ml), which exceeds the expected tissue concentrations and highlights the need for more sensitive platforms. Accordingly, a high-sensitivity ELISA was used to successfully quantify anti-TNF in all fistula samples as well as in paired rectal biopsies obtained as mucosal control tissue for assay validation. An additional methodological limitation relates to variability in sampling relative to anti-TNF dosing, which may influence measured serum concentrations and derived tissue penetration metrics. As sampling was not standardised to dosing intervals, the time since last anti-TNF administration varied between patients, resulting in heterogeneity in systemic drug exposure at the time of tissue collection. To address this, secondary analyses focused on tissue penetration expressed as fistula-to-serum ratios, providing a proportional assessment of local tissue drug exposure relative to contemporaneous systemic levels. While this approach helps mitigate the impact of variable serum concentrations driven by dosing timing, residual pharmacokinetic variability cannot be fully excluded.

The small sample size represents the principal limitation of this study, which reduces the statistical power and limits generalizability. However, as one of the first investigations to directly measure matched serum and fistula anti-TNF concentrations in pfCD, these findings provide an important proof-of-concept for the role of tissue pharmacokinetics in assessing the therapeutic response. Although ELISA has been widely validated for serum drug quantification, it lacks formal standardisation for tissue homogenates [16], which may influence reproducibility, as noted in recent methodological reviews [22]. Nevertheless, ELISA remains an accessible and scalable platform compared with more specialised analytical methods, and pending further validation, may offer pragmatic utility for incorporating tissue-level drug assessment into personalised treatment strategies for pfCD.

In conclusion, this proof-of-concept study demonstrated that anti-TNF drug levels in perianal fistula tissue are measurable and correlate with serum levels. Higher fistula-to-serum ratios, reflecting improved tissue penetration, tended towards better clinical and radiological outcomes, although the statistical significance was limited by the small sample size. In line with calls to optimise anti-TNF therapy through personalised approaches [29,30], these findings support further investigation in larger prospective cohorts to define clinically relevant tissue-level drug targets and clarify the role of tissue pharmacokinetics in guiding integrated medical–surgical management strategies for perianal fistulising Crohn’s disease.

## Acknowledgements

The authors thank Paul Bassett (Statsconsultancy Ltd) for his valuable expertise in statistical analysis and interpretation. The authors would also like to acknowledge Clair Bewshea of the Exeter Clinical Laboratory for assistance with detecting anti-TNF drug levels.

Tissue samples were provided by the Imperial College Healthcare NHS Trust Tissue Bank, funded by the National Institute for Health Research (NIHR) Biomedical Research Centre based at Imperial College Healthcare NHS Trust and Imperial College London. The views expressed are those of the author(s) and not necessarily those of the NHS, the NIHR or the Department of Health. Human samples used in this research project were obtained from the Imperial College Healthcare Tissue Bank (ICHTB). ICHTB is supported by the National Institute for Health Research (NIHR) Biomedical Research Centre based at Imperial College Healthcare NHS Trust and Imperial College London. ICHTB is approved by Wales REC3 to release human material for research (22/WA/0214), and the samples for this project, R20025, were issued from sub-collection reference number SMT_PT_20_001.

### Conflicts of interest

S.A. received European Crohn’s Colitis Organisation Research Grant in 2022 and has received conference attendance support from AbbVie, Johnson & Johnson, Dr Falk and Ferring.

L.H. received conference attendance support from Falk and The Leona M and Harry B Helmsley Charitable Trust.

J.L.A. received speaker fees from Abbvie, Takeda, Pfizer and Janssen. He have received travel grants and support to attend meetings from Lilly, Tillotts Pharma, Takeda and Celltrion.

N.P. has received research grants from AstraZeneca, Bristol Myers Squibb, CCUK, Celltrion Healthcare, Helmsley Charitable Trust, Pfizer and Takeda; received personal fees from AbbVie, Allergan, AstraZeneca, Bristol Myers Squibb, Celgene, Eli Lilly, Galapagos, GlaxoSmithKline, Janssen, Pfizer, Takeda and Roche; served as a speaker or on the advisory board for AbbVie, Allergan, AstraZeneca, Bristol Myers Squibb, Celgene, Dr Falk, Galapagos and Vifor; and serves on the data safety monitoring board for AstraZeneca and Bristol Myers Squibb.

S.J.C. received research grants for investigator-initiated projects or trials from Abbvie, Agency for Clinical Innovation, Amgen, BMS, Chiesi, Celltrion, DrFalk, Ferring, Janssen, Medical Research Future Fund, Pfizer, South Western Sydney Local Health District, Sydney Partnership for Health, Research and Enterprise, Takeda and The Leona M and Harry B Helmsley Charitable Trust. Consulting fees from Abbvie, Amgen, BMS, Celltrion, Eli Lilly, Ferring, GSK, Janssen, Organon, Pfizer and Takeda. Payment honoraria from Cornerstones Health, Dr Falk, Ferring, Janssen, Sandoz, Sydney IBD School and Takeda. Conference attendance support from Dr Falk, Sandoz and Takeda. Stock options in SONIC Healthcare.

P.T. received speaker fees and/or advisory board for Takeda, J&J, Tillots, Ferring and Falk.

A.H. served as consultant, advisory board member or speaker for AbbVie, Arena, Atlantic, Bristol Myers Squibb, Celgene, Celltrion, Falk, Galapogos, Lilly, Janssen, MSD, Napp Pharmaceuticals, Pfizer, Pharmacosmos, Shire and Takeda. She also serves on the Global Steering Committee for Genentech.

For the remaining authors, there are no conflicts of interest.
